# Comparison of spotlighting monitoring data of European brown hare (*Lepus europaeus)* relative population densities with infrared thermography in agricultural landscapes in Northern Germany

**DOI:** 10.1371/journal.pone.0254084

**Published:** 2021-07-09

**Authors:** Katharina Sliwinski, Egbert Strauß, Klaus Jung, Ursula Siebert

**Affiliations:** 1 Institute for Terrestrial and Aquatic Wildlife Research, University of Veterinary Medicine Hannover, Foundation, Hannover, Germany; 2 Institute for Animal Breeding and Genetics, University of Veterinary Medicine Hannover, Foundation, Hannover, Germany; Sichuan University, CHINA

## Abstract

A successful wildlife management requires monitoring. Including non-scientific volunteers into monitoring actions is a common way for obtaining long-term and comprehensive data. Hunters present a valuable target group as they are spread out nationwide in Germany and additionally, they provide a know-how regarding game species. Since 1990s, various German hunting associations established monitoring programs and motivated hunters to join, in order to record population sizes of huntable game species under standardized census methods. The aim of this study was to compare instructed hunters performed spotlight counts of European brown hares with thermography in three federal states (Lower-Saxony, Saxony-Anhalt, North Rhine-Westphalia) in 2015–2018 in Northern Germany. Therefore, we modelled the number of hares counted by both methods with the associated observed area. Moreover, we performed repeated thermographic counts in selected areas and performed distance sampling to test the deviations of estimated population densities within a short time period. Repeated infrared thermographic counts on three consecutive nights show a coefficient of variation from 6.6% to 15.5% with deviations of 2.2–2.7 hares per 100 ha, while the method of distance sampling reveals minor deviations of 0.9–1.7 hares per 100 ha and a coefficient of variation from 3.1–7.4%. The coefficient of variation value between spotlight and infrared thermographic count lies between 0 to 21.4%. Our model confirmed no significant differences between the European brown hare density estimations based on a spotlight count and an infrared thermographic count on the following night. The results provide insight into the dimension of the error margin of density estimations performed by spotlight counts. Therefore, we recommend to take possible counting errors into account and to ideally perform repeated counts to assess the error margin for each counting site. This would help for example to quantify the uncertainty in the calculation of mortality rates. Additionally, our results show that monitoring data generated by instructed hunters can provide reliable and valid data, if implemented and conducted in a standardized scientific way.

## Introduction

Accurate wildlife population estimations play a major role in understanding the temporal population dynamics, in evaluating management effectiveness, and in detecting changes in the first place [1, 2]. Especially with regard to biodiversity loss, monitoring programs are an essential conservation activity for detecting declining species, therefore, they present a key part in wildlife conservation [3–5].

Embedded as a conservation tool, declined monitored species should trigger management intervention [6]. As important as a plan of action is a monitoring program provides accurate data. Otherwise monitoring data would be strongly limited in its interpretation [7–9]. In order to test the quality of monitoring data, an evaluation which assesses its validity is useful [1].

The European brown hare *Lepus europaeus* is a characteristic species of the European agricultural landscape. However, since the 1970s a decline has started across Europe, mostly caused by the intensification of agriculture [10–13]. The development, especially the intensity of the decline, varies widely depending on the region [14, 15]. The early population data of hares are based on hunting bags [11, 16]. Hunting bags may provide trends on a large scale but may not be representative of real changes, they provide only inaccurate data with low effort [17–21]. In order to provide analysis in regard to the overall decline as well as to develop and assess management strategies, long-term monitoring data with a high accuracy are necessary [22, 23].

Spotlight counts using point or line transects have become an established standard method for assessing relative abundance, growth and mortality rates and habitat use patterns of hares. The method assumptions require inter alia that the population distribution counted in the illuminated area equals the distribution of the not illuminated area [22, 24, 25]. Several studies deal with the validity of the method and the significance of the resulting population estimations [26–28] as well as with the challenge of interpreting the results [29]. Various factors have an impact on the reliability of a count, including the visual capacity of the observer, the visibility due to environmental factors (e.g. weather conditions), or the distribution of hares and their visibility (e.g. contrast between hare and ground) [30, 31]. Until now, a validity check of spotlight counts on Lagomorphs (with multiple consecutive counts within a short period) has shown diverse coefficients of variation from 13.3% to 32.6% [32–35]. The method of spotlight counts was designed to be applicable by instructed laymen and therefore, be an effective tool in citizen science projects [31].

Including hunters into monitoring programs is beneficial for several reasons, they are representated in agricultural areas nation-wide and they have expertise regarding game species [22].

Since the 1990s, diverse hunting associations of different federal states have initiated hunters to count European brown hares with spotlights on their hunting grounds (e.g. “Wildtiererfassung Niedersachsen”, “Niederwildzensus Baden-Württemberg”). In 2001, the German wildlife information system “WILD” (Wildtier-Informationssystem der Länder Deutschlands) was founded with the aim to merge available data on population sizes of huntable game species, inter alia the spotlighting data on European hares from the monitoring programs of the federal states [36].

Until now, no validation of the WILD data has been carried out, while there are several options available to evaluate the method of spotlight counts.

Infrared cameras are widely used to detect wild animals and to estimate their population size—from small to medium sized to large animals [27, 37]. The decisive factors limiting thermography are weather condition and vegetation cover, which reduce the effective visible width [38, 39]. Comparative studies on census accuracy of spotlight counts and infrared counts reveal that thermography shows a higher population density due to improved detection [40]. The high acquisition costs of initial equipment has been inhibiting thermography usage for large-scale monitoring by a large number of participants [27].

Distance sampling has been established as an effective method for wildlife estimation in open habitats as the results are highly precise [41]. Furthermore, it resolves a substantial part of problems, which occur when direct counts are performed, as it models variation in species’ detectability [41]. The method was found to be suitable for estimating Lagomorphs. So far, distance sampling has been applied on Cape hare *L*. *capensis* in South Africa [42], brown hare *L*. *europaeus* [43–45], mountain hares *L*. *timidus*, Irish hares *L*. *timidus hibernicus* in Europe [46, 47] and *Oryctolagus cuniculus* in Spain [48]. A detailed review of different methods to assess hare abundance by Langbein et al. [24] supports distance sampling, as it leads to robust results, especially when the method is applied during night time [44].

The aim of the present study is to compare spotlight counts performed by instructed hunters using thermography. We assume that infrared thermography is a suitable technique for testing spotlight counts on hares as we expect that both methods produce similar population density estimations. As thermography enables a more precise estimation due to technical advantages the value has a higher accuracy than the density estimation based on the spotlight count. Furthermore, we test consecutive counts within a very short time window (< 3 days) by thermography and distance sampling in selected areas in order to determine deviations in time delay counts.

## Materials and methods

### Study area

**Fig 1:**
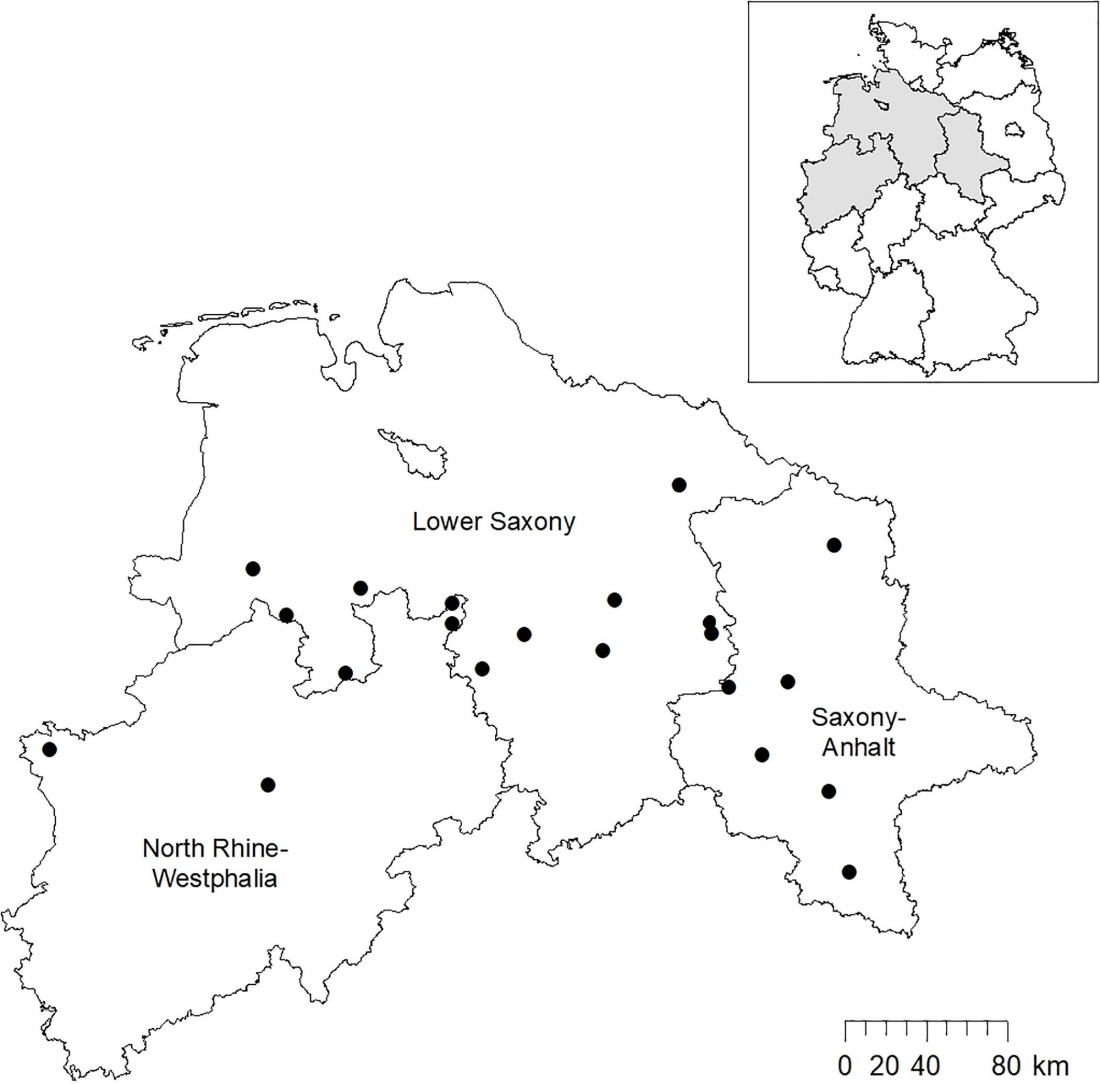
Overview of the study areas. Lower Saxony (11 reference areas), North-Rhine Westphalia (4 reference areas) and Saxony-Anhalt (6 reference areas).

The surveys took place between 2015 and 2018 in 22 reference areas, spread over three Federal States; 12 located in Lower-Saxony, 4 in North Rhine-Westphalia and 6 in Saxony-Anhalt (Fig 1). Each study area was inspected at daytime and mapped in ArcGIS Desktop 10.6.1 (ESRI Inc., US), capturing crop types, the growth state and areas with limited visibility during the counts. Fields with a growth state higher than 30 cm were excluded as they would be considerably limited regarding the detectability of hares. During the census at night, another inspection of non-visible surfaces was necessary, as some sight restrictions (e.g. shadow areas due to ground waves) are only identifiable at night. As our counting took place after harvest and before vegetation growth, we had the best possible detectability conditions. The track was recorded by a GPS Garmin 64 during the counts. Reference areas had an average of 824.3 ha of huntable area, an average of 274.1 ha of illuminated area and a mean track length of 21.6 km.

Although Lower Saxony, North Rhine Westphalia and Saxony-Anhalt are neighbouring Federal States, the topography and the agriculture structures differ widely. The western Federal States of Lower-Saxony and North Rhine Westphalia show a high structural diversity in agricultural landscapes with lots of hedges, edge structures and small fields. Saxony-Anhalt on the other hand, shows huge fields with lower structural diversity with narrow windbreaks and lines of trees with some bushes. The structural changes in agriculture are caused by the different political systems of the agricultural economy in East and West Germany before the reunification [25].

### Spotlight count method in the monitoring program “WILD”

The hunters of each reference areas conduct line-transect spotlight counts in spring (Feb-March) and autumn (Oct-Dec) in order to collect data for hare density estimations. The counts are performed 1–2 hours after sunset. In general, weather conditions do not affect the hare distribution, with the exception of extreme weather conditions such as mist, fog, heavy rains and strong winds. During their counts, standardized protocols are filled in, including time and date, weather conditions and a tally sheet for detected hares on each transect with a list of different possible land use forms. The line transects in a reference area should be representative regarding the agricultural crops. The conditions for standardized spotlight counts are a reference areas, which shows a minimum size of 400 ha, an illuminated area of a minimum 200 ha and a forest proportion of less than 30%, as hares prefer open lands for feeding at night. All line transects of a reference area should give a total track length of ca. 20 km. The hares are counted from a moving car, which is driving at about 10–25 km/h. The effective illuminous range of the spotlight should be about 150 m. In Lower Saxony, a handheld spotlight (Conrad Electronics, 55 W) is used by holding it in a right angle from the passenger window. The hunters of North Rhine-Westphalia use the Conrad Electronics spotlight and comparable models. In Saxony-Anhalt, a spotlight (modified Bosch, 55 W) is mounted through a suction cup to the passenger window in a right angle. During the procedure, hares are detected with the naked eye by their silhouette and their reflecting Tapetum lucidum. If an individual cannot be identified, binoculars are used. It is recommended to perform the counts two times per season respectively. A third count is recommended if the difference between the first and the second count exceeds 30% of the first count [36].

During autumn, the suitable time for counts is after the main harvest of sugar beet and maize but before the high growth of catch crops such as white mustard or phacelia. Each count should be undertaken two hours after sunset when hares have their maximum activity [49].

The spotlight count method of Pegel [50] is applied in Lower Saxony, while the method of Ahrens [51] is applied in Saxony-Anhalt. Both methods are similar and only differ in usage of different spotlight types and in calculation of the illuminated area.

The calculation of illuminated areas in Lower-Saxony is done with ESRI ArcGIS, which is necessary as it is characterized by very structured landscapes and many sight restrictions. The effective strip width of 150 m is projected into a map, and sight restrictions are removed from the illuminated area calculation.

The hunters of North Rhine Westphalia illuminate–if possible—their entire open agricultural land of their hunting ground. Due to small structured fields and a tight field track system this procedure is possible in North Rhine-Westphalia. Thus, the illuminated area equates the open area of the hunting ground.

The landscape structure of Saxony-Anhalt is characterized by large field block sizes and fewer hedges (which means only marginal sight restrictions). Therefore, the method of calculating the illuminated area by multiplying the effective strip width of 150 m with the transect route was established. This way, the calculation of the illuminated area with ArcGIS has become rather unnecessary in eastern hunting grounds.

For our comparison, European hare relative population density on the basis of spotlight counts was calculated as:

$$relative\;population\;density=\frac{N_{hares}*100}{illuminated\;area\;(ha)}$$

In order to achieve a convergence in our data set we also digitized our selected reference areas of Saxony-Anhalt in ArcGIS.

As part of the study design, a scientist took part as an observer during the spotlight count without offering comments or corrections. We considered this necessary to check whether the spotlight counts were performed in our study in same manner as before in the monitoring program. The implementation of a spotlight count was documented according to standardized protocol. These hunters had a max. driving speed of 25 km/h along representative line transects, holding the handheld spotlight in a very calm right angle with an optimal illumination on the surface and at depth, noting the non-visible areas into a protocol, starting the spotlight count two hours after sunset. In this way spotlight counts can be completed in two to two and a half hours.

### Infrared thermographic count

The comparison of spotlight counts by using thermography was conducted on the following night after the spotlight count, taking the same track and starting at the same time. We used the infrared camera system Nyxus Bird with an integrated laser range finder. Thermographically observed hares were identified by their silhouettes and movement. For increasing detectability, the observer was standing on the loading area of a pick-up truck. The observer was secured in a harness and attached to a rack on the loading area. Because of this, both sides of the track could be observed. The speed of 5–10 km/h was depending on the track and weather conditions as well as the topography of the study area. Therefore, thermographic counts were performed between three till four hours. The infrared thermographic count was only performed under optimal weather condition, as foggy or rainy conditions reduce the probability of detection.

European hare relative population density on the basis of infrared thermographic counts was calculated as:

$$relative\;population\;density=\frac{N_{hares}*100}{observed\;infrared\;area\;(ha)}$$

In order to use an infrared thermographic count in our testing procedure, we conducted multiple thermographic counts. In autumn 2018, we measured the variation of population densities in reference areas in Lower Saxony on three consecutive nights starting as the spotlight counts 1–2 hours after sunset. To avoid observer bias, the same person conducted all infrared thermographic counts [52]. In order to avoid a learning bias, the person practiced the observation and detectability of hares with infrared thermography before starting the survey.

During the planning of our study design, we considered a fluctuation of population densities between different counting nights. To ensure that the detection of hares with a spotlight is not biased [53, 54], we excluded a simultaneous implementation of both methods on the same vehicle. The method of two separate vehicles driving behind each other was also excluded as animals can show flight response during census [55]. Moreover, hares can start to flush even before a vehicle approaches [56]. Furthermore, the driving speed of both methods differed between 10–15 km/h. Additionally, the infrared thermographic count contained regular stops for accurate hare detection, while the spotlight count required a constant driving.

### Distance sampling

Distance sampling is an effective tool for the estimation of wildlife density besides spotlight and infrared thermographic counts. Once an individual is detected, its distance needs to be measured in a right angle to the line-transect before it starts to move. As the camera used in our study incorporates a laser range finder, the data set for wildlife estimation by infrared thermographic count and distance sampling is collected at the same time. For each distance measurement the vehicle needed to stop. When the hares fled, we measured the distance between the observer and the spot where the individual had been sitting in the first place.

With the distances of the measured object and line transect length, distance sampling calculates a “detection function” which estimates the likelihood of the detection of an animal. Furthermore, an effective strip width is calculated. This way, wildlife density and accuracy is being determined. All data preparation and analyses were conducted in R 3.5.3 [57]. The statistical models were conducted in the R package ‘Distance’. We used the uniform key with cosine adjustment, the halfnorm key with cosine adjustment, the half normal key with hermite polynomial adjustment, and the hazard rate key with simple polynomial adjustment—models as recommended in Thomas et al. [58]. The best model and adjustment term was selected on the basis of Akaike´s information criterion (AIC). We conducted Kolmogorov-Smirnov, Cramer-von Mises, and chi-square tests to check for overall goodness of fit, as implemented in the R-package “Distance”.

If the detections of individuals do not decrease with distance but rather show a peak, far away animals have possibly flushed because of the approaching vehicle. Due to an enlargement of the first interval in the model, the so called “evasive movement” can be resolved for better model fit of the data [45, 59]. This procedure can underestimate the density if hares are avoiding the proximity to roads. In order to remove biased data from closed proximity to the transect, a left truncation presents an alternative processing by truncating the data near zero. This procedure may lead to an overestimation of hares, as there will be too many observations further away [41]. As recommended, we also right truncated 5–10% of the objects detected at the largest distances [41].

### Analytical and statistical procedure

All data preparation and analyses were conducted in R 3.1.2 [57]. The aim of the first step was to check the deviation of estimated densities during consecutive infrared thermographic counts within three days (no. of hares per 100 ha observed area). For a better comparison we consulted the coefficient of variation, which represents the ratio of the standard deviation to the mean. The coefficient of variation was calculated between nights per each reference area. We choose five reference areas for consecutive night counts in Lower-Saxony (reference area: B, Le, V, H, U). Additionally, in three of these reference areas (reference area: B, Le, V) distances of the hares individuals were measured to apply distance sampling. For a comparison of thermography and distance sampling we calculated the Spearman’s rank correlation coefficient.

For the comparison between spotlight counts performed by hunters and thermography, one scientist took participation as an observer during the spotlight counts in order to ensure that the scientific standard. We calculated the standard deviation between the population densities of a spotlight and an infrared thermographic count and calculated the coefficient of variation between the nights per reference area as well as the Spearman’s rank correlation coefficient.

Additionally, we performed a generalized linear mixed model (GLMM) in order to compare the spotlight count and the consecutive infrared thermographic count under the assumption of different distribution (e.g. Poisson and negative binomial distribution) and compared by means of the AIC. Wheather variables were not recorded and therefore not included into the model. In total, n = 22 locations were observed by spotlight and thermographic counts. Two-sided hypotheses in the GLMM model were tested, i.e. there was no prior expectation for one method to have higher counts than the other one. As response variable, the counted number of hares was used, and the method as well as the observed area as the fixed effects. The reference area was integrated as a random effect.

## Results

### Variation of relative population densities based on infrared thermographic counts in three consecutive nights

Within five consecutive infrared thermographic counts in five reference areas (reference area: B, Le, V, H, U) 1,715 hares were counted in an accumulated area of 7,420.5 ha.

Two areas were represented with a medium-low hare relative population density (mean: 13.5 hares per 100 ha), two areas with a medium-high sized density (mean: 22.2 hares per 100 ha), while one area showed a high density (40.2 hares per 100 ha). The deviation between all three infrared counts in five areas ranged from 2.1 hares per 100 ha to 5.0 hares per 100 ha. The standard deviation was similar in each reference area (2.2–2.7 hares per 100 ha observed area). The coefficient of variation of repeated infrared counts within an area varied between 6.6–15.5% (S1 Table).

### Comparison of relative population densities based on infrared thermographic counts with relative population densities based on distance sampling in three consecutive nights

In three of five reference areas (B, Le, V) enough observations were conducted in order to apply distance sampling. The density estimations with distance sampling in three selected areas showed quite similar densities compared to the infrared count (Spearman’s rank correlation coefficient, r = 0.96). The smallest hare number for distance analysis was counted in reference area V with 69 individuals, while the highest was counted in reference area B with 262 individuals (S1 Table). The standard deviation of each region is lower than in infrared counts (0.9–1.7 hares per 100 ha observed area). The coefficient of variation within an area varied between 3.1–7.4% (S2 Table). As recommended by Buckland et al. (2001), we performed a cut of 5% of the furthest sightings and therefore right truncated our data to 350 m. Observation frequencies were generally low near the transect line—a peak is visible further away instead.

### Comparison of relative population densities based on spotlight counts performed by hunters with relative population densities based on infrared thermographic counts

**Fig 2:**
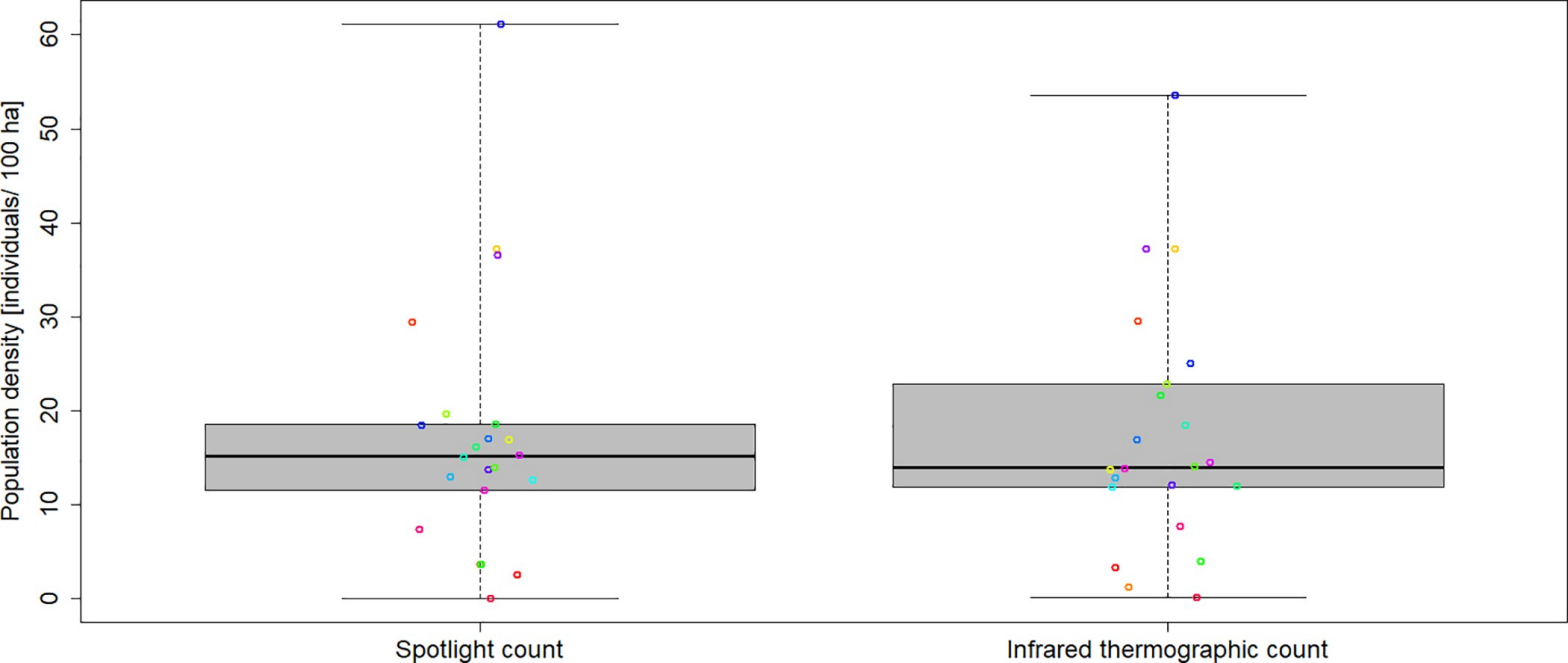
Estimated European hare population densities. No. of hares per 100 ha observed area on the basis of 22 spotlight counts and 22 infrared counts in three federal states (Lower Saxony, Saxony-Anhalt, North Rhine-Westphalia) of Germany.

Since the thermography presents a reliable method, it is suitable to be applied for a comparison with spotlight counts. On the following day of each of the 22 spotlight counts an infrared count was performed (S3 Table). 1,000 hares were counted in total in an area of 6,031 ha with spotlights, while 2,062 hares were counted on a total area of 12,379 ha with an infrared camera (S3 Table). During both counts the same tracks were used, each having a total length of 474 km. The relative hare population density calculated on the basis of a spotlight count varied from extreme low values between 0 hare per 100 ha in a reference area in Saxony-Anhalt to 61.2 hares per 100 ha in a reference area in Lower-Saxony. The density estimation by infrared thermographic counts varied between 0.1 hare per 100 ha in Saxony-Anhalt to 53.6 hares in Lower-Saxony (Fig 2).

The coefficient of variation varied from 0 to 21.4% (excluding two reference areas with extreme low hare abundances, S3 Table).

**Fig 3:**
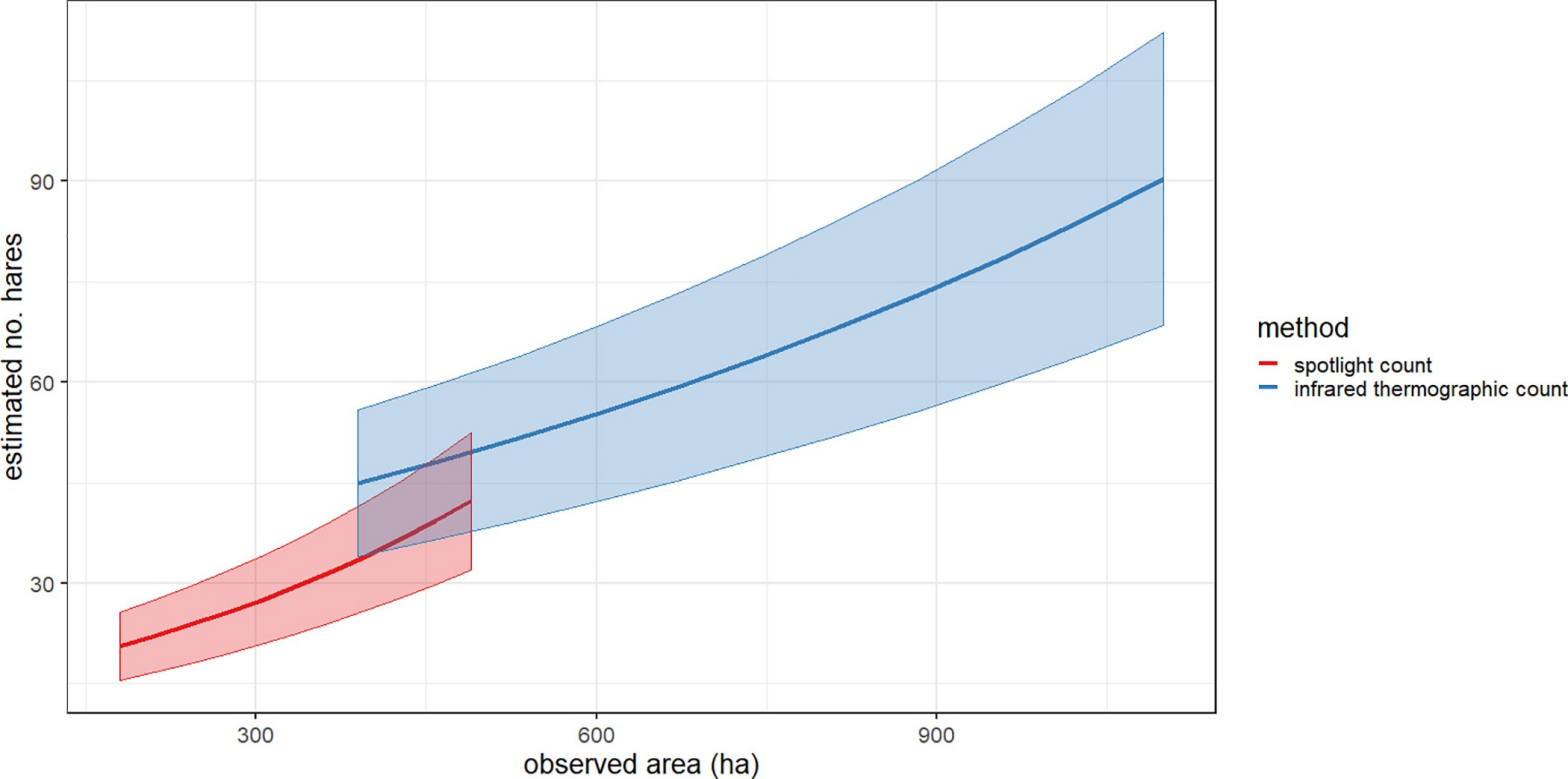
Estimated numbers of counted European hares. On the basis of 22 spotlight counts and 22 infrared counts in three federal states (Lower Saxony, Saxony-Anhalt, North Rhine-Westphalia) of Germany with a fitted Poisson GLMM curve and a 95% confidence bands.

Our GLMM showed no significant difference between both methods (p = 0.8). Nevertheless, the trend line of the infrared count is continuously higher than the trend line of the spotlight count (Fig 3).

## Discussion

The hare population densities deviated +/- 3 hares per 100 ha and revealed a coefficient of variation from 6.7% up to 15.5% on the basis of consecutive infrared thermographic counts. Only few studies focus on repeated hare counts within a short time period. Our maximum coefficient of variation is comparable to a previous study on Lagomorphs in the Jordanian steppe [32], where repeated counts were performed over nearly a month. We assume that the habitat structures influence the results of hare census [24, 60]. A homogenous, flat habitat might lead to an equal hare distribution with less fluctuation in multiple density estimations, while a more heterogeneous habitat (like an alpine environment) lead to an unequal aggregation of hares and sight restrictions, which leads to more diverse census results [60]. Moreover, our study areas are also characterized by several tree cover and hedgerows (< 30%), which can prevent or limit animal detection and constitute the limitation of spotlights and thermal imaging cameras. Some studies performed many repeated counts on hares over medium-term, where wind speed and direction lead to significant variations in the numbers of counted individuals [61].

Our distance sampling data set shows less observation of hares near the centerline, but rather a peak of sightings in subsequent intervals, which might be caused by evasive movement. Incomplete detection of individuals leads to a negative bias in abundance estimates [62]. Three models could be improved by grouping the data, enlarging the first interval and a right truncation. Five models showed best model fit with only right truncated data. While one model showed best model fit with left (100 m) and right truncation. It needs to be considered that the AICs with different truncations are not appropriate to compare as these models use different data. Therefore, we chose the best model based on shape criterion and GOF. Left truncation lead mostly to a better model but increased the density considerably.

In contrast to our study, the distance data set of Petrovan et al. [44] shows less absence data in the first interval. In order to improve the model we performed a full left truncation and a grouping of our data and enlarged the first interval. Nonetheless, the best model fit was achieved by applying only a right truncation.

The assumption of an accurate distance measurement in a perpendicular angle was achieved by measuring distances with a laser range finder after stopping the vehicle in a right angle to the initial position of the animal.

While planning this survey, we were aware that using determined routes (field paths) trespasses the assumption of distance sampling [41]. Previous studies demonstrated a bias due to the use by public roads i.e. for white tailed deer [63]. Since random choosing of transects in cultivated areas from the ground is not possible, we selected minor roads, as previous studies received acceptable results by this study design [47]. Preferably, we used minor field tracks which are continuously representative in agriculture landscape. Due to the fact that field paths are not public, they are mainly undisturbed by conventional traffic. Thus, we believe that our choice of transects enables a tolerable study design in distance sampling. As the density estimations of our chosen distance sampling models are comparable to our simultaneous infrared count and the associated area calculation, distance sampling is a suitable method for hare density estimations in scientific studies of selected areas in German Lowlands.

Large-scale citizen science studies require many study areas with many participants and an easy and cost-effectively applicable method. Therefore, the method of distance sampling is not applicable for hunters due to its relatively complex implementation. Additionally, this method has its limits with low abundances, as the minimum of 60–80 sightings should be guaranteed for fitting the detection function [41]. On the other hand, distance sampling can be quite difficult to apply in areas with extreme high hare abundances as in our experience, hares start to flush already when the vehicle approaches. That’s why the exact distance measurement of every individual becomes challenging to impossible.

When the hunters complied with the guidelines of accurate spotlight counts, they provided data which guaranteed a scientific standard. Other citizen science projects also confirm hunters as a suitable target group as their collected data strongly correlates with professional techniques [64].

Due to the necessity of a large set of participants in a citizen science monitoring program, it should be taken intro account that an observer bias and a visibility bias is non-avoidable, which can vary up to 30% in spotlight surveys [40]. Additionally, it must be pointed out that there are challenges for an efficient monitoring program, such as data quality and the retention of citizen participants over long periods [65]. The quality can be improved by a personal contact with the hunter, regular visits in their study area and regular assessments of their method implementation (i.e. every 3–5 years) in order to prevent mistakes and negative bias. Especially the necessity of complying with a scientific standard at all times needs to be successfully communicated.

The spotlight count and the associated infrared thermographic count have a coefficient of variation from 0 up to 21.4%, which is slightly higher compared to our previous, consecutively performed infrared thermographic counts. These fluctuations of the estimated population densities between consecutively performed counts may be influenced by habitat [66], which is why we have different coefficients of variation. Additionally, abiotic factors such as moon phase, wind, rainfall and temperature may have an influence, which may lead to different hare estimations [32, 34, 35, 61].

A comparison of spotlight counts and forward-looking infrared (FLIR) counts in a previous study on white-tailed deer also showed no significant difference between both methods, although it did show that the best overall observability was achieved with FLIR [67]. Focardi et al. [27] observed no difference in the results between spotlighting and thermal imaging of species containing a tapetum lucidum. Our results support these results, as the method of spotlight counts and infrared thermographic counts show no significant differences.

A basic assumption of spotlight counts is a regular spatial hare distribution. However, we know from previous studies that the results do not completely comply with the theory [68]. Hares rather show group formations, which are distributed irregularly. Especially agricultural practise leads to the avoidance of certain areas [68]. Due to potential high changes in spatial hare distribution, 2–3 repeated spotlight counts should be conducted within an interval of 7–10 days for solid density estimations. Despite multiple counts per season, excessive deviations may occur based on random or systematic errors [69].

The above-mentioned results outline the complexity of analysing and interpreting of the spotlight data. However, the spotlight count is the best method for an overall study including many study areas and volunteers. Whereas, distance sampling and thermography are suitable to be applied in studies with a low sample size conducted by scientists. Especially the thermography is highly beneficial as it is non-invasive, providing minimal disruption and distress to animals [70].

One downside of the study design is the fact that the dimension of measurement error of each method is unknown and cannot be reliably estimated in surveys, as the total number of individuals in a given area remains unknown [39]. It is difficult to predict which of the applied methods was the most useful for estimating the most likely true hare density, but we are convinced that spotlight counting is the best census method when comparing total expense and large scale feasibility in a citizen science project. As described in previous literature [50, 51], it is crucial that the participants strictly meet the standard of spotlight counts. Furthermore, for the interpretation of spotlighting data, its assumptions of the theory need to be taken into account.

Based on our results, the method of spotlight counts is an appropriate procedure and applicable by instructed hunters to generate valid estimations of European hare populations in visible open agricultural habitats in the Northern Lowlands of Germany.

A wide use of thermal imagery was limited in the past because of the high acquisition cost [27]. As thermography devices have become more affordable, the circumstances may change in the near future. At present, a lot of hunters acquire infrared devices. Therefore, it can be considered as a new tool in future monitoring programs [71]. The application of different thermal imaging equipment leads to similar detectability in wildlife [72]. However, a precondition for the usage of thermographic devices are generally applicable scientific guidelines and a correct recording of the detected area for each camera model.

## Conclusions

Obtaining valid data on wildlife population densities and developments is crucial for a successful and efficient management and for conservation of wildlife resources. Within our comparison with thermography the method of spotlight counts performed by hunters (who complied the standardized guidelines) produced reliable and comparable estimates of European hare densities in open agricultural habitats in the Northern Germany Lowland federal states Lower-Saxony, Saxony-Anhalt and North Rhine-Westphalia. Although the infrared count is not significantly different from the spotlight count it seems to be slightly more precise. Nevertheless, both methods are appropriate in order to receive long-term population data. In the case of monitoring the spotlighting method is the best alternative due to its suitable, logistically feasible and low-cost application. However, the method needs to be adapted to the regional conditions. If performed under standardized implementation, modifications of spotlight counts allow supra-regional comparison. Therefore, a coherent implementation of the method is of particular importance. The cornerstones of this effective monitoring program require a detailed preparation, including a scientific establishment of reference areas, an accurate training in scientific work for hunters as well as providing motivation for long-term participation. The spotlight count method as described in the present study is only suitable for open, flat agricultural land. The method reaches its limits in hedgerow-dominated habitats or forest habitats due to reduced visibility. However, point counts or photo trapping may provide valid results in these areas, but a verification for comparable density data to linear spotlight counts would be required first. It is unlikely to find only one superior method for estimating European hare population densities for all habitat types.

## Supporting information

S1 Table Numbers of total counted hares with infrared thermography. Population density (PD), standard deviation (SD) and associated coefficient of variation (CV) of European hares of three repeated infrared thermographic counts conducted in Lower Saxony, Germany autumn 2018.
(DOCX)

S2 Table Distance sampling based density estimations. Distance sampling based density (D) per 100 ha of European brown hares in three reference areas in Lower-Saxony, Germany, for autumn 2018, and combined infrared survey. SD = standard deviation; CV = coefficient of variation; 95% LCL = lower confidence limit; 95% UCL = upper confidence limit.
(DOCX)

S3 Table Estimated European hare population densities on the basis of spotlight and infrared thermographic counts. The calculation basis for the density estimations of both methods is given as the total number of counted hares and the associated covered area of 22 study areas in three federal states in Germany between 2015–2018.
(DOCX)

S4 Table General linear mixed model explaining estimated number of hares with the method of spotlight and thermographic count. (DOCX)

S1 File (CSV)

S1 Data (R)
