# Supplementary material for: Comparison of spotlighting monitoring data of European brown hare (Lepus europaeus) relative population densities with infrared thermography in agricultural landscapes in Northern Germany
Source: PLoS One. 2021 Jul 9;16(7):e0254084. doi: 10.1371/journal.pone.0254084 (PMC8270206; doi:10.1371/journal.pone.0254084)
Supplement: S1 Table — Population density (PD), standard deviation (SD) and associated coefficient of variation (CV) of European hares of three repeated infrared thermographic counts conducted in Lower Saxony, Germany autumn 2018. (DOCX) [file pone.0254084.s001.docx]

**S1 Table. Numbers of total counted hares with infrared thermography.**

| reference area | count number | total number of counted hares | observed area (ha) | PD (no. Hares per 100 ha observed area) | mean | SD | CV |
| --- | --- | --- | --- | --- | --- | --- | --- |
|  |  |  |  |  |  |  |  |
| B | 1 | 231 | 620.4 | 37.2 | 40.2 | 2.7 | 6.6 |
| B | 2 | 256 | 620.4 | 41.3 |  |  |  |
| B | 3 | 262 | 620.4 | 42.2 |  |  |  |
| Le | 1 | 72 | 596.7 | 12.1 | 14.1 | 2.2 | 15.5 |
| Le | 2 | 83 | 596.7 | 13.9 |  |  |  |
| Le | 3 | 98 | 596.7 | 16.4 |  |  |  |
| V | 1 | 82 | 326.1 | 25.1 | 24.1 | 2.6 | 10.8 |
| V | 2 | 69 | 326.1 | 21.2 |  |  |  |
| V | 3 | 85 | 326.1 | 26.1 |  |  |  |
| H | 1 | 51 | 394.2 | 12.9 | 12.9 | 2.7 | 7.8 |
| H | 2 | 55 | 394.2 | 14 |  |  |  |
| H | 3 | 47 | 394.2 | 11.9 |  |  |  |
| U | 1 | 121 | 536.1 | 22.6 | 20.2 | 2.7 | 11.6 |
| U | 2 | 107 | 536.1 | 20 |  |  |  |
| U | 3 | 96 | 536.1 | 17.9 |  |  |  |

Population density (PD), standard deviation (SD) and associated coefficient of variation (CV) of European hares of three repeated infrared counts conducted in Lower Saxony, Germany autumn 2018.
