# Supplementary material for: Comparison of spotlighting monitoring data of European brown hare (Lepus europaeus) relative population densities with infrared thermography in agricultural landscapes in Northern Germany
Source: PLoS One. 2021 Jul 9;16(7):e0254084. doi: 10.1371/journal.pone.0254084 (PMC8270206; doi:10.1371/journal.pone.0254084)
Supplement: S2 Table — Distance sampling based density (D) per 100 ha of European brown hares in three reference areas in Lower-Saxony, Germany, for autumn 2018, and combined infrared survey. SD = standard deviation; CV = coefficient of variation; 95% LCL = lower confidence limit; 95% UCL = upper confidence limit. (DOCX) [file pone.0254084.s002.docx]

**S2 Table. Distance sampling based density estimations.**

| reference area | Key-function | Model | D | 95% LCI | 95% UCI | SD | CV |
| --- | --- | --- | --- | --- | --- | --- | --- |
| B | Hazard rate | Right truncation, 350m | 35.6 | 24.6 | 51.7 | 1.1 | 3.1 |
| B | Hazard rate | Right truncation, 350m | 37.7 | 29.0 | 48.9 |  |  |
| B | Hazard rate | Right truncation, 350m | 37.4 | 27.6 | 50.8 |  |  |
| Le | Hazard rate | enlarged interval | 11.5 | 0.6 | 20.7 | 0.9 | 7.4 |
| Le | Hazard rate | enlarged interval | 13.3 | 0.7 | 23.2 |  |  |
| Le | Hazard rate | Right truncation, 350m; Left truncation, 80m | 12.8 | 0.7 | 22.3 |  |  |
| V | Hazard rate | enlarged interval | 25.7 | 17.0 | 38.7 | 1.7 | 6.3 |
| V | Hazard rate | Right truncation, 350m | 27.0 | 16.1 | 45.2 |  |  |
| V | Hazard rate | Right truncation, 350m | 29.1 | 18.4 | 46.0 |  |  |

Distance sampling based density (D) per 100 ha European brown hares in three reference areas in Lower-Saxony, Germany, for autumn 2018, and combined infrared survey. SD= standard deviation; CV= coefficient of variation; 95% LCL = lower confidence limit; 95% UCL = upper confidence limit.
