# Supplementary material for: Comparison of spotlighting monitoring data of European brown hare (Lepus europaeus) relative population densities with infrared thermography in agricultural landscapes in Northern Germany
Source: PLoS One. 2021 Jul 9;16(7):e0254084. doi: 10.1371/journal.pone.0254084 (PMC8270206; doi:10.1371/journal.pone.0254084)
Supplement: S3 Table — The calculation basis for the density estimations of both methods is given as the total number of counted hares and the associated covered area of 22 study areas in three federal states in Germany between 2015–2018. (DOCX) [file pone.0254084.s003.docx]

**S3 Table. Estimated European hare population densities on the basis of spotlight and infrared thermographic counts**

| **federal state** | **reference area** | **number of counted hares with spotlight** | **illuminated area with spotlight** | **estimated population density on spotlight count basis** | **number of counted hares with thermography** | **observed area with infrared camera** | **estimated population density on infrared thermographic count basis** | **variation of coefficient** |
| --- | --- | --- | --- | --- | --- | --- | --- | --- |
| Lower-Saxony | A | 53 | 180.0 | 29.4 | 117 | 397.2 | 29.5 | 0.2 |
| Lower-Saxony | B | 119 | 319.6 | 37.2 | 231 | 620.4 | 37.2 | 0 |
| Lower-Saxony | H | 31 | 190.9 | 16.2 | 54 | 449.1 | 12.0 | 2.1 |
| Lower-Saxony | Ho1 | 33 | 218.7 | 15.1 | 73 | 394.2 | 18.5 | 14.3 |
| Lower-Saxony | Ho2 | 33 | 262.1 | 12.6 | 47 | 394.2 | 11.9 | 3.9 |
| Lower-Saxony | Ho3 | 34 | 262.1 | 13.0 | 51 | 394.2 | 12.9 | 0.2 |
| Lower-Saxony | L | 150 | 245.0 | 61.2 | 358 | 667.5 | 53.6 | 9.4 |
| Lower-Saxony | Le | 47 | 339.8 | 13.8 | 72 | 596.7 | 12.1 | 9.6 |
| Lower-Saxony | Li | 70 | 191.8 | 36.6 | 188 | 505.2 | 37.2 | 1.2 |
| Lower-Saxony | U | 39 | 246.2 | 15.3 | 78 | 536.1 | 14.5 | 3.8 |
| Lower-Saxony | W | 21 | 181.3 | 11.6 | 50 | 358.6 | 13.9 | 12.8 |
| Lower-Saxony | We | 14 | 188.0 | 7.4 | 55 | 712.0 | 7.7 | 2.8 |
| Northrhein-Westphalia | Bo | 36 | 360.6 | 16.9 | 54 | 393.6 | 13.7 | 14.8 |
| Northrhein-Westphalia | Bw | 45 | 228.9 | 19.7 | 55 | 240.9 | 22.8 | 10.3 |
| Northrhein-Westphalia | I | 83 | 488.3 | 17.0 | 62 | 367.9 | 16.9 | 0.4 |
| Northrhein-Westphalia | K | 49 | 267.4 | 18.5 | 123 | 489.2 | 25.1 | 21.4 |
| Saxony-Anhalt | Ad | 10 | 393.0 | 2.5 | 24 | 711.9 | 3.3 | 19.5 |
| Saxony-Anhalt | Ba | 8 | 217.1 | 3.7 | 8 | 655.9 | 1.2 | 72.1 |
| Saxony-Anhalt | Do | 52 | 327.0 | 14.0 | 155 | 1096.5 | 14.1 | 0.5 |
| Saxony-Anhalt | F | 12 | 337.2 | 3.6 | 28 | 700.3 | 4.0 | 7.4 |
| Saxony-Anhalt | Gs | 61 | 327.1 | 18.6 | 178 | 820.7 | 21.7 | 10.9 |
| Saxony-Anhalt | Z | 0 | 259.7 | 0 | 1 | 876.8 | 0.1 | 141.4 |
| **total** | **22** | **1000** | **6031.8** |  | **2062** | **12379.1** |  |  |

The calculation basis for the density estimations of both methods is given as the total number of counted hares and the associated covered area of 22 study areas in three federal states in Germany between 2015-2018. On the basis of both methods the coefficient of variation is given.
