# Supplementary material for: Comparison of spotlighting monitoring data of European brown hare (Lepus europaeus) relative population densities with infrared thermography in agricultural landscapes in Northern Germany
Source: PLoS One. 2021 Jul 9;16(7):e0254084. doi: 10.1371/journal.pone.0254084 (PMC8270206; doi:10.1371/journal.pone.0254084)
Supplement: S4 Table — (DOCX) [file pone.0254084.s004.docx]

**S4 Table. General linear mixed model explaining estimated number of hares with the method of spotlight and thermographic count.**

| Variable | Estimate | SE | p-value | Sign |
| --- | --- | --- | --- | --- |
|  |  |  |  |  |
| Intercept | -2.153 | 0.242 | <2e-16 | *** |
| Method: thermographic count | 0.009 | 0.039 | 0.8 |  |
